# When all computers shut down: the clinical impact of a major cyber-attack on a general hospital

**DOI:** 10.3389/fdgth.2024.1321485

**Published:** 2024-02-16

**Authors:** Benyamine Abbou, Boris Kessel, Merav Ben Natan, Rinat Gabbay-Benziv, Dikla Dahan Shriki, Anna Ophir, Nimrod Goldschmid, Adi Klein, Ariel Roguin, Mickey Dudkiewicz

**Affiliations:** ^1^ Hospital Administration, Hillel Yaffe Medical Center, Hadera, Israel; ^2^ Ruth and Bruce Rappaport Faculty of Medicine, Technion – Israel Institute of Technology, Haifa, Israel; ^3^ Surgical Division, Hillel Yaffe Medical Center, Hadera, Israel; ^4^ Pat Matthews Academic School of Nursing, Hillel Yaffe Medical Center, Hadera, Israel; ^5^ Division of Obstetrics and Gynecology, Hillel Yaffe Medical Center, Hadera, Israel; ^6^ Risk Management Department, Hillel Yaffe Medical Center, Hadera, Israel; ^7^ Division of Pediatrics, Hillel Yaffe Medical Center, Hadera, Israel; ^8^ Division of Cardiology, Hillel Yaffe Medical Center, Hadera, Israel

**Keywords:** computers in medicine, computer security, cyberattack, healthcare system resilience, patient electronic file, ransomware, safety

## Abstract

**Importance:**

Healthcare organizations operate in a data-rich environment and depend on digital computerized systems; thus, they may be exposed to cyber threats. Indeed, one of the most vulnerable sectors to hacks and malware is healthcare. However, the impact of cyberattacks on healthcare organizations remains under-investigated.

**Objective:**

This study aims to describe a major attack on an entire medical center that resulted in a complete shutdown of all computer systems and to identify the critical actions required to resume regular operations.

**Setting:**

This study was conducted on a public, general, and acute care referral university teaching hospital.

**Methods:**

We report the different recovery measures on various hospital clinical activities and their impact on clinical work.

**Results:**

The system malfunction of hospital computers did not reduce the number of heart catheterizations, births, or outpatient clinic visits. However, a sharp drop in surgical activities, emergency room visits, and total hospital occupancy was observed immediately and during the first postattack week. A gradual increase in all clinical activities was detected starting in the second week after the attack, with a significant increase of 30% associated with the restoration of the electronic medical records (EMR) and laboratory module and a 50% increase associated with the return of the imaging module archiving. One limitation of the present study is that, due to its retrospective design, there were no data regarding the number of elective internal care hospitalizations that were considered crucial.

**Conclusions and relevance:**

The risk of ransomware cyberattacks is growing. Healthcare systems at all levels of the hospital should be aware of this threat and implement protocols should this catastrophic event occur. Careful evaluation of steady computer system recovery weekly enables vital hospital function, even under a major cyberattack. The restoration of EMR, laboratory systems, and imaging archiving modules was found to be the most significant factor that allowed the return to normal clinical hospital work.

## Introduction

It seems possible to imagine the modern world without computers. Medical care systems have advanced globally for many years. Currently, several of these systems are now paperless. Digitalization has significant advantages and allows access to all records in an online and real-time format. However, the dependency of healthcare organizations on digital computerized systems and the data-rich environment of these systems create vulnerability to cyber threats. Indeed, hacks and malware are a major concern in the healthcare sector (1).

Healthcare systems may be exposed to various types of cyber threats, which can be categorized as internal or external. Internal threats arise from inappropriate access to sensitive data by internal proxies, while external threats arise from external agents exploiting the vulnerability of healthcare information systems. External threats include data breaches, denial-of-service attacks, cybersquatting, critical infrastructure failure/breach, and cyberterrorism. Cybercriminals aim to steal or corrupt personalized health information, which in turn can harm patients, healthcare organizations, healthcare networks, and even the entire nation (2). The motivation is usually financial but may also be terrorism.

As healthcare systems become increasingly reliant on digital systems to deliver care, healthcare organizations’ readiness to manage critical infrastructure failure/breach is crucial for the continuity of care and patient safety. Analyzing healthcare organizations responses to malfunctioning computerized systems can provide new insights that may be useful in the management of such future events.

Multiple cyberattacks have been reported in the medical literature, most of which describe the technical aspects of the cyberattacks or their impact on a single hospital service/department (3–5). However, the impact of hacks and malware on entire healthcare organizations remains under-investigated.

In what appears to be one of the largest medical cyberattacks in Israeli history, the Hillel Yaffe Medical Center (HYMC) major networks were hit with a major ransomware attack, by an unknown hacker group. As a consequence, the whole hospital was locked out of its entire digital systems. The ransom attack was declared as a major national incident. During the following 8 weeks, the hospital gradually resumed its regular activities, and total computer recovery was achieved.

This study aimed to identify and evaluate the direct and indirect impact of the cyberattack on the hospital's clinical activities and its organizational workflow. We explored the significance of the restoration of electronic medical records (EMR) and radiology archiving modules on normal hospital work.

### Setting

HYMC is a general and acute care university teaching hospital. It is a non-profit, governmental-owned organization with a 546-bed capacity, including 35 different inpatient wards and ambulatory clinics. The hospital provides a wide range of medical services to a diverse population with various medical conditions. It serves a referral population of ∼500,000 people. It is the only hospital in the area located 50 km from the nearest tertiary hospital. The hospital employs ∼3,000 workers.

The hospital's information management is comprehensively computerized across all organizational levels, including clinics, administration, logistics, and communications. All hospital functions were disrupted. Computerized systems include core platforms, such as EMR (Chameleon, Elad Software Systems, Tel Aviv, Israel) connected to medical devices, admission, discharge, and transfer (ADT) and the main enterprise resource planning (ERP) system (ERP’s Namer, SAP, Ra’anana, Israel), logistics (Mazor, SAP), Radiology Information System & Picture Archiving and Communication System (RIS/PACS, Phoenixville, PA, USA), IVF software (Eve Pro, OBG Soft, Israel), laboratory system (AutoLab and Softov, Softov Medical Systems, Kfar Saba, Israel), and a widespread communications network including Microsoft Outlook, an employee attendance system, a surveillance camera network, and additional security systems. The hospital is connected to the national community health patient record (HPR) interface—“Eitan” software, a computerized platform unique to Israel, which enables the sharing of online secure patient information between different healthcare providers and health insurance companies. In addition, the hospital management board uses a wide range of reports and data systems for obtaining online information on hospital activity status, control, supervision, and decision-making.

There are also about 100 different niche systems working across the hospital. In general, all organizational documentation is performed without paper documents and includes clinical treatment management, patient clinical data, measures, and follow-up, computerized physician order entry (CPOE), operating room (OR) module, patient education, laboratory, different protocols and reports, and communication between departments and services. Furthermore, during the COVID-19 period, the hospital added and upgraded remote e-work such as e-consultation services, remote home access, and other services.

### Description of the cyberattack

On October 13, 2021, the Hillel Yaffe major networks were hit by a ransomware attack, known as DeepBlueMagic, by an unknown hacker group. All computer systems, at all levels of the hospital, were locked, without the option to log in. This denial-of-service attack locked completely the entire computer network by blocking access to most computers and encrypting most of the external and internal servers.

This particular type of ransomware attack utilized an innovative type of encryption. It is particularly dangerous because it manages to circumvent protection tools installed in the system to protect it. The attackers utilized a legitimate tool such as Microsoft BitLocker to disable all computers. The malware locks the system, and the hackers may demand a ransom to release it, holding the information, and potentially the lives, of the hostage patients (1).

The attack was immediately recognized by an administration server and reported to the National Ministry of Health and the National Cyber Council. HYMC immediately locked the Internet access to prevent further damage, essentially blocking access to EMR, laboratory and imaging results, and other vital digital tools.

As a result of the attack, no access was available to any hospital computer. The staff did not even know which patients were scheduled for appointments nor the patient list on their respective wards. It influenced every aspect of medical services, both inpatient and outpatient. This included the loss of some documentation of patient data, EMRs, inpatient lists, information on upcoming outpatient appointments, laboratory services and reporting of results, imaging availability, and more. Also, the entire online staff communication system using intranet and e-mails came to a halt.

## Methods

For the present manuscript analysis, the week of the DeepBlueMagik ransom attack was defined as week *X*. We performed two analyses (1). Data were collected during the 8-week period following the attack and compared to data from the parallel period in 2019. Data regarding the year 2020 were not examined due to the COVID-19 pandemic limitations (2). We compared the activity in the weeks following the cyberattack to the activity in the week of the attack.

The study was approved by the local IRB Committee, 0035-22-HYMC. The collected data did not include any patient personal or medical information. Research information was secured and saved in a suitable software for which access was limited to the study's investigators.

The case study method was used to describe the hospital's response to the massive cyber ransom attack. Data collection was based on the Ministry of Health information systems and activity reports extracted from HYMC information systems. All data collected manually during the attack were completely returned to the hospital computer systems. Data included visits to emergency departments (ED); the number of hospital admissions; hospital clinical activities, including urgent cases and elective procedures; the occupancy rate of pediatric, surgical, internal, and obstetric departments; and the number of visits to outpatient clinics.

### Statistical analysis

The empirical analysis consisted of two steps. For this study, to maximally neutralize the potential impact of the COVID-19 pandemic and holidays, we collected data regarding hospital activities during the parallel 8-week period in 2019. This data served as a control for comparison. First, we compared the means of the two periods (2019 vs. 2021 across the postattack weeks), using the Mann–Whitney non-parametric test for two independent samples, that come from the same population to determine whether two sample means are equal or not. A complementary incidence rate ratio (IRR) analysis examined whether the rates remained unchanged across the two periods, that is, whether drops in the number of admissions, procedures, and outpatient visits were similar across different hospital departments and external services. Following the abovementioned analysis, the Mann–Kendall trend test was used to investigate the week-by-week difference, that is, the difference during each week through the duration of the attack (weeks *X*–*X* + 7) (6–8). Sen's non-parametric slope measures were added. A graphical exposition was presented to show the change across weeks from large to no difference. For this analysis, we looked at different clinical time series, which covered hospital activity in all aspects, specifically, emergency room (ER) admissions, direct admissions to hospital departments, emergency admissions to hospital departments, total occupation rates, surgeries, cardiovascular catheterizations, number of births, and imaging services, i.e., CT, MRI, and x-rays.

## Results

### Immediate actions taken following the cyberattack

The top priority was to keep the patients safe. National news aired the issue, and prehospital emergency services were instructed whenever possible not to refer mild and moderate trauma patients to the HYMC emergency department. This was a critical decision that reduced the number of patients entering the hospital.

An immediate decision was made to stop all non-life-saving procedures, and many surgeries had to be rescheduled. Some patients had to be referred to other centers for various treatments. A decision was made to halt all elective surgeries and invasive procedures until guaranteeing that the hospital ventilators, all ICU monitors, operation rooms, and catheterization laboratory infrastructures have not been damaged.

During the first week, there was significant activity disruption throughout the hospital, both for the patients and for the healthcare staff. These disruptions included reverting to manual processes, e.g., reporting blood test results, paper documentation, writing and executing medical orders by handwriting, canceling outpatient appointments, and elective admissions and most surgical procedures (Table 1).

**Table 1:** Challenges arising following the cyber attack.

Service affected	Sequela	Adaptation
Emergency room (ER)	A gradual decrease in visit numbers	The media reported cyberattack calling patients to turn to other ERs; ambulances referred patients to other ERs
Elective procedures	All elective procedures (hospital-wide) were postponed	Risk stratification was done on all elective procedures. Potentially complicated surgeries were referred to other hospitals
Loss of electronic medical records (EMR)	No historical data on patients from outpatient clinics, or previous hospitalizations	Within 2–3 days, a full set of Word formatted documents were available for ER visits (admission or discharge), interventional procedures, birth certificates, ward follow-up, and discharge letters
Labs	No previous or current laboratory results were available for patients in the ER or admitted	Only critical labs were done. Tubes were tagged using handwritten stickers and were sent manually to the laboratory. The results were first reported on the phone and within a few days were printed and delivered manually to the sending department.
Blood bank services	No historical data on the blood type of recurring patients. Risk of mislabeling patient blood type with increased risk of compromise to patient safety	Blood product transfusions were decreased to a minimum. Extra caution was implemented in rare cases when a blood product transfusion was needed.
Imaging	No remote access and historical images available	Selected imaging was done. Images were photographed using smartphones for radiological assessment
Decision-making	Change in policies for sending labs, imaging, and/or admission	Senior physicians or senior residents were placed to allow for better triage, deferring unneeded tests and preferring ambulatory care, whenever possible. Complicated cases (with potential risk for complications that will require complicated surgery or blood–product transfusion were deferred or referred to other medical centers. Admission was selected for low-risk patients who could not be discharged
Elective cesarean sections		Elective cesareans were allowed after risk management and case selection
Newborn identification	No digital authentication or digital birth certificates	Was done with double verification by two midwives, written manually on stickers and tagged to newborns and mothers
Outpatient clinics	The scheduled patient list was not available. No history or previous encounter data	Patients were treated based on “whoever arrives.” Following encounters, patients were triaged: “uncomplicated” cases were referred to other clinics. Patients were encouraged to bring hard copies of their medical records
Archiving data	No data archiving. All data were manually written on paper	All data were assembled in the patient's binders. The first page included the order of papers from the admission letter, diagnosis list, follow-up, labs, fetal heart monitoring, etc. Every paper included a place for the date and hour and the physician's signature. Later all documents were scanned into the patient's EMR

### Computer system recovery

The system recovery efforts began from day 1 after the attack, first including the HPR interface, by distributing laptops with a secured cellular Internet connection to the national “Eitan” system, to restore the hospitalized patient health history. Laptops were distributed in the different wards and connected directly to printers (as there was no printer network) to allow clinical follow-up, the production of a Word document for discharge letters, and the writing of follow-up.

The first decisions taken by the local IT and national cyber experts were to build a completely new network for restoring the data, disconnected from the Internet (with security layers), and installing the entire systems from scratch (without information restoration). New servers were installed, and a separate network was formed with new IP addresses for all the equipment. To reduce the occurrence of similar mishaps in the future, the local IT conducted password replacement in all systems, an examination, and elimination of permissions that were unnecessary. The same software were installed separately, and when possible, the data were restored and scanned for malware. After the imported data were installed in the new systems, each restored database was approved and only then allowed to be used. The local IT aimed to create a “new” and clean system. New layers of security and updated information security systems, such as antivirus, with the latest signatures were implemented to enable connection to the Internet and external connectivity to suppliers.

The laboratory system (LabOs) was restored in the first week after the attack (*X* + 1), enabling the necessary work processes involved in laboratory testing. The ATD system was repaired in the middle of the second week following the attack (*X* + 2), which restored the ability to properly register the hospital's population demography and patient flow. The second week (*X* + 2) also included the restoration of the radiology module (RIS/PACS), which allowed image archiving, editing, distribution, and storage of patient radiological data. The administration and operation platform was restored after the fourth week (*X* + 4) and enabled proper administrative ability. The EMR system, including all patients’ histories that were saved in backup systems before the attack, was reinstalled in the fourth week (*X* + 4), restoring the standard routine of electronic patient documentation and clinical data management. Finally, following 8 weeks (*X* + 8), intranet and e-mail communication were restored. Week by week, partial recovery of the computer systems allowed the hospital's administration to carefully and gradually return to normal activity, along with meticulous evaluation of changes associated with this process.

### Main study findings and outcomes

#### Drop in activity in 2021 compared with 2019

Descriptive statistics for the main time series across the two defined periods, followed by a non-parametric test of difference are presented in Tables 2, 3. Across the different time series, no statistical difference was found in the number of heart catheterizations (*U* = −0.950, *p* = .370; *U* = 0.306, *p* = .766), as well as in the number of births and outpatient visits. In all other time series, the two periods differed, where the mean level of all other evaluated activities in the postattack period was lower in comparison to 2019. The complementary IRR test results indicated that the proportion of women admitted to the obstetric department and of children admitted to the pediatric department remained similar, regardless of the attack [the 2021–2019 ratio was 1.46 95% CI: (1.39,1.53); 1.10 95%-CI: (1.03,1.67), for women and children, respectively]. That is, in 2021, the proportion of women and children for all hospital admissions was higher, notwithstanding the attack.

**Table 2:** Comparison between 2019 and 2021 across various time series; weekly means.

	2019 mean ± SD	2021 mean ± SD	*p*-value
1. Total number of emergency room (ER) admissions^a^	2,479.8 ± 117.0	2,084.2 ± 284.2	.001
2. Total number of emergency direct admissions to hospital departments	661.8 ± 32.2	552.0 ± 99.8	.016
3. Hospital occupancy (percent of beds occupied)	83% ± 5%	64% ± 9%	<.001
4. Total number of ambulatory visits	6,640 ± 1,242.5	5,801 ± 1,163.4	.056
5. Births	86.3 ± 10.6	78.6 ± 5.6	.056
6. Total number of surgeries	243.1 ± 62.2	143.3 ± 102.4	.020
7. Heart catheterizations	26.9 ± 5.7	27.6 ± 6.5	.766
8. Total number of ambulatory imaging services	2,525.5 ± 198.8	2,013.6 ± 560.9	.016

^a^ One excluded COVID admission.

**Table 3:** Analyses of the return to routine across various hospital indicators.

	Mann–Kendall trend test, *Z*	*p*-value	Sen’s slope, 95% CI
Total number of emergency room (ER) admissions	−2.81	.002	−105.75,[−124.55,−86.95]
Total number of emergency admissions to hospital departments	−1.77	.038	−26.63, [−45.43,−7.83]
Hospital occupancy (percent occupied beds)	−0.52	.301	−0.01, [−18.81,18.79]
Total number of ambulatory visits	−0.31	.377	−70.83, [−89.63,−52.03]
Births	1.15	.126	1.92, [−16.88,20.72]
Total number of surgeries	−0.52	.301	−10.02, [−28.82,8.78]
Heart catheterizations	−0.94	.174	−1.74, [−20.54,17.06]
Total number of ambulatory imaging services	−2.19	.014	−188.08, [−206.88,−169.28]

Comparisons are based on differences across weeks *X* to *X* + 8.

**Figure 1:**
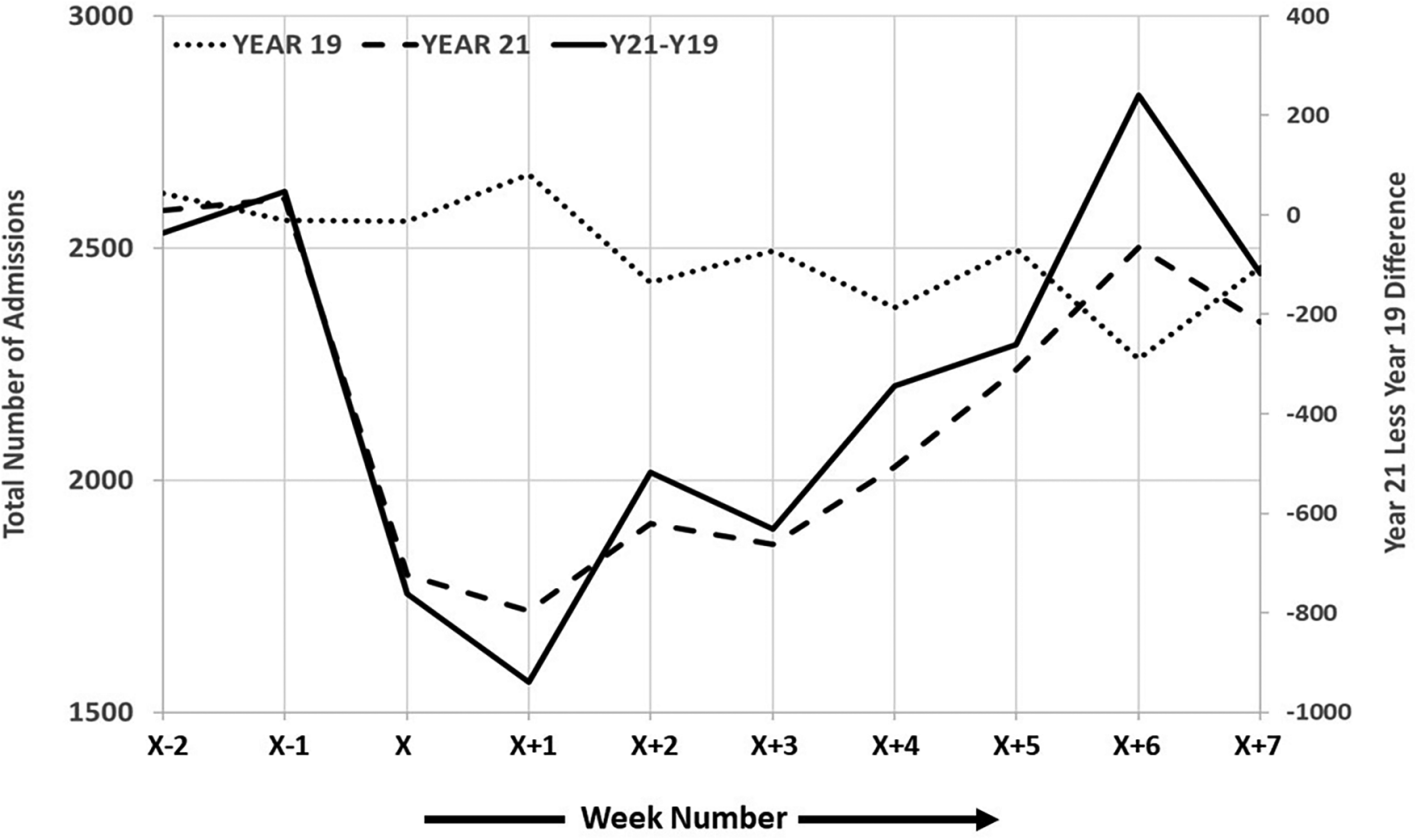
Graphical representation of the two periods with regard to total ER admissions.

The weekly number of ER admissions during 2019 and 2021 was analyzed, and the parallel weeks in the two periods were compared. Although administrative protective interventions were performed immediately, in certain time series, its impact was observed only as of *X* + 2. The weekly differences were highly correlated with the 2021 level, as in 2019 the trend was relatively flat, as shown in Figure 1. The indication is that the trend took place mainly from week *X* + 2 to week *X* + 6. Assessment of the differences between 2021 and 2019 would be caught up to zero, or no difference. We found significant Mann–Kendall test results (*p* = .002) and a significant (*p* < .05) negative Sen's trend. The number of admissions dropped to slightly higher than 1,500 in week *X* + 2 of 2021, compared with ∼2,500 in regular weeks. Based on Sen’s slope calculation, we state that the difference was caught up by an average of 100 cases during the postattack period.

During the week of the ransomware attack, there was an overall small decrease in the number of deliveries, which remained stable during the 8 weeks that followed, representing a small drop compared to the average number of deliveries in the respective weeks in 2019 (86.3 ± 10.6 vs. 82.8 ± 11.2, 2019 and 2021, respectively). Visits to the obstetric ER demonstrated a decrease compared to *X*−1 and *X*−2, with a small difference compared to the average visits per week in the retrospective period in 2019. This remained stable up to *X* + 8 (234.5 ± 19.5 vs. 225.7 ± 25.1, *p* = 0.00, 2019 and 2021, respectively).

#### Return to activity compared to the week of the cyberattack

A final analysis is presented in Table 4. In this analysis, percent changes in numbers were calculated for different hospital activity measures. We found the actual change for these series and tested whether a significant change has occurred between the event (week *X*) and stages in the return to normal activity (week *X* + 2, PACS + RIS; week *X* + 4 and Chameleon system recovery; week *X* + 5, full capacity). We estimated a trend across the 8 weeks that followed the event and found that aside from ambulatory visits, cardiovascular catheterizations, and ambulatory imaging services, the other six series showed a significant decrease in percent differences with respect to the earlier weeks of the event.

**Table 4:** Tests of return to normal by significant events.

Percent change between weeks →	Trend	*X* + 2/*X*−2	*X* + 4/*X*−2	*X* + 6/*X*−2	*X* + 2/*X*	*X* + 4/*X*	*X* + 6/*X*
Hospital activity measure							
Emergency room (ER) admissions	−.04***	23.1%	18.2%	−0.9%	−6.2%	−13.0%	−39.4%
Emergency hospital admissions	−.05**	31.5%	25.9%	4.9%	6.0%	−1.8%	−30.7%
Regular hospital admissions	−.09**	46.3%	18.5%	−2.8%	42.0%	12.0%	−11.0%
Occupied beds	−.04*	35.2%	22.9%	17.3%	15.9%	0.0%	−7.2%
Ambulatory visits	−.02	35.7%	35.7%	16.7%	26.0%	26.0%	4.1%
Births	−.04*	35.4%	18.8%	−3.1%	33.5%	16.5%	−6.1%
Surgical procedures	−.06*	50.1%	24.1%	−0.6%	36.1%	2.7%	−28.8%
Heart catheterizations	.002	26.6%	19.1%	24.5%	15.9%	7.3%	13.4%
Ambulatory imaging services	−.05	40.0%	26.7%	−3.3%	5.3%	−15.8%	−63.2%
*T*-test results		12.49***	12.29***	1.64	3.59*	0.84	−2.36*

* *p* < 0.05.

** *p* < 0.01.

*** *p* < 0.001.

## Discussion

In recent years, laptops and tablets have become as common in healthcare settings as stethoscopes, and for the young physician and nurse generation, working without computers seems impossible. The present study aimed to identify and evaluate the direct and indirect impact of the DeepBlueMagic cyberattack, which involved a complete computer and network shutdown, on the hospital’s clinical activities and its organizational workflow. There was a need to install new servers and create a new network, and the first functions to return were the EMR and laboratory module and the imaging module.

The decision of a new network was recommended by the National Cyber Council, and the order of software installation was based on which systems were most crucial for the fast and safe return of clinical treatment and management of the patients in the hospital. There was a need to prioritize, and each system was released for use only after extensive checks and verification of all data. This took time but building the base was important to create a stable system.

Our results demonstrated that the attack impact differed according to the type of hospital activities. Most clinical activity was impacted apart from cardiovascular catheterizations, ambulatory outpatient visits, and ambulatory imaging services. The restoration of EMR, laboratory systems, and imaging archiving modules was found to be the most significant factor that allowed the return to normal clinical hospital work.

On the day of the cyberattack, all non-vital elective procedures were canceled. As expected, a drop was detected in all elective hospitalizations. Emergency services were asked to detour the hospital. There was a sharp drop detected in all admissions in the week of the attack. However, there was a complete return to the baseline hospitalization numbers in the internal medicine wards at week *X* + 7. Yet, surgical emergency admissions did not return to the baseline even after week *X* + 8. The total number of hospitalizations in the obstetrics and gynecology wards remained unchanged during the study period. It is easy to explain why there has not been a decline in gynecological interventions, as procedures such as caesarian sections and abortions cannot be canceled.

We demonstrated a significantly sharp drop in the number of admissions to the pediatric and psychiatric emergency wards in the week of the attack, accompanied by a consistent increase in these admissions after the first 7 days. This was regardless of interventions made by the hospital administration and the system recovery status. We assume this patient population, opted to return to the nearest hospital in any case. The gradual return to surgical activities was restarted at week *X* + 2. In the beginning, the hospital administration considered performing surgeries in cases where the consequences of a delay, outweighed the risk of the operation, according to the clinical judgment of the chief of the department.

In such a crisis, medical decision-making was crucial. The simple decision to send labs or admit/release to ambulatory care was based not only on guideline recommendations but also on risk stratification. This included, on the one hand, being aware of the potential damage to patients by human errors, delay of treatment, or suboptimal care and, on the other hand, enabling medical care when required. For example, the need for blood transfusion carries a special risk of human error (when taking the blood, tagging the tubes, forwarding them to the labs, retrieving a matching blood product, and transfusing it in the patient). Patients who were at high risk for complications during an intervention and who might need imaging services or interdisciplinary care were also postponed if possible or referred elsewhere.

Early indications suggest that the current “DeepBlueMagic,” gained initial access by exploiting remote access software—a known Pulse Secure VPN vulnerability. The exploitation of network infrastructure is consistent with previously reported DeepBlueMagic activity, an unsurprising revelation given that many ransomware operators favor tried-and-tested exploits to acquire user credentials and/or gain privileged access to victim networks. It is also common for ransomware operators to terminate processes and services associated with backup and security tools, to evade detection and further thwart recovery and any application servers, and to ensure that files are not locked open.

This incident should act as yet another reminder as to why it is important to ensure that network infrastructure devices, all too often deployed and forgotten about, are included in robust patch management programs. Defenders will be reliant on the need to detect behavioral activity, both prior to the encryption phase, such as unusual user logon activity and privilege escalation, and during the encryption phase, such as the unexpected execution of these utilities or anomalous disk and file operations.

Moreover, despite the published recommendations and guidelines, there seems to be no single solution for managing cyberattacks, due to the complexity of such events and the differences between healthcare systems (9, 10). For example, a large regional orthopedic service in Ireland that was subjected to an attack decided to utilize inter-hospital transfers based on radiological hard copies and secure messaging systems and in some situations using the WhatsApp application (11). However, in the absence of clear formal or at least worldwide-accepted regulatory processes, it may create ethical concerns regarding patient privacy (12). For example, the Israeli data security laws prohibit such use. Furthermore, in comparison with evidence-based medicine, the solutions seem to be intuitive in many situations. Thus, during the major “WannaCry” attack, the National Health System (NHS) in England published a “Cyber Handbook” that reviewed the lessons learned and security standards and described ways to prepare for future events (13). However, this publication does not detail local cyber response activities in any depth. In addition, during the “WannaCry” attack, the authors reported no difference in the total level of activities across all the system trusts, but a statistically significant 6% drop in total admissions in the “infected” hospitals. However, this attack lasted only 1 week, and no single hospital-related volume analysis was performed (14).

As opposed to previous studies (14–21), the main effort in the current study was to evaluate the timing of problem identification and the different software installation and to investigate the decision-making processes with regard to different outcomes of hospital activities. The temporary conversion to a manual documentation process allowed the continuation of vital services and data collection. The hospital had to form a new network with new servers installed. The IT priority was to assist in the most important services: HRP, laboratories, and imaging. Some clinical services such as catheterizations were not influenced, but some took longer to recover. Some, as a result of rescheduling and waiting for a proper backup system to restore data, were destroyed by the attack.

According to a recent US Government interagency report, there have been 4,000 daily ransomware attacks on average since early 2016 (a 300% increase compared to the 1,000 daily ransomware attacks reported in 2015; US Government Interagency Guidance Document). The amount of data stored, which consists of financial information, health details, social security information, and others, its sensitivity, and the growing dependence of medical providers on technology have made hospitals a viable target for cyberattacks (16).

It is important to emphasize that this ransom attack was considered and treated by our organization as an emergency event. As part of the Israeli reality, which requires facing various emergencies, HYMC, similar to other Israeli healthcare organizations, created and constantly maintains preparedness for a variety of anticipated emergencies, one of which is a cyberattack/computerized systems overall shutdown. The components of an emergency preparedness and response program include planning, training, simulations, information management, communication, development of response, and contingency plans.

This is not the first time that a healthcare institution has been targeted, and undoubtedly not the last, given that some may consider them a soft target. The disruption caused by these financially motivated cyberattacks could result in a loss-of-life situation by delaying or preventing critical care. Several papers describe ways to prevent, recover, and analyze such attacks (22, 23). These studies emphasize the importance of monitoring computer and application use continuously in an effort to detect suspicious activities and identify and address security problems before they cause harm. There is a need to ensure adequate system protection by correctly installing and configuring computers and networks that connect them and to ensure more reliable system defense by implementing user-focused strategies, including simulation and training on the correct and complete use of computers and network applications. Finally, organizations need to respond adequately to and recover quickly from ransomware attacks and take action to prevent them in the future.

One limitation of the present study is that due to the retrospective design of this study, there were no data regarding the number of elective internal care hospitalizations that were considered crucial. Another major limitation is that this study describes a single-center experience with its own conclusions, which are not necessarily relevant to every institution in future cyberattacks.

Being prepared and simulating a cyberattack, when no computers function, may assist every institution in understanding the strengths and weaknesses they might have. Many daily life clinical examples can be detected with such a simulation, for example, how to locate the different forms such as consent forms and order sheets, how to connect to printers not through a network, how to send blood and other body fluid tests, how to receive the result and record them, and how to look at a CT scan or even a simple x-ray of a fractured bone—if there is no network or the option to burn a CD as there is no network. How can someone see a patient in the ambulatory outpatient clinic, when the physician is “blinded” to any clinical note, imaging test, laboratory, or pathology results or has no access to the ECG in the hospital’s database? Simulation in each institute can find many additional points and detect weak areas, that one might face; but today—in the computer era—we do not think about it. Table 5 summarizes some additional points.

**Table 5:** Main lessons for future events.

Checklist for a cyber incident	
Pre-established mechanisms for communication with healthcare teams	
Return to paperwork: pre-prepared sets of blank, hard-copy medical records in each department to enable uninterrupted charting and documentation	Templates for all scenarios should be readily accessible, with hard copies to allow workflow. This is important for patient safety and to ensure that all relevant patient details are recorded for later archiving
Staff training in the use of manual documentation and procedures for cyberattacks or other computer system losses (videos and simulations of work processes)	
Simulation of cyberattack	To detect the current situation in each organization
Safety first	Patient safety above all and considering the risk/benefit of each step
Decision-making	Senior physicians were allocated to every station to cope with both medical and ethical dilemmas. The decision for ambulatory care, referring to other hospitals, or even using the imaging services/labs was not based solely on medical recommendations but also took into consideration the complexity and risk of every evaluation needed
Laptop backup	To have enough laptops for urgent distribution
Communication and teamwork	e-mails or contact lists originally stored on hospital IT platforms were no longer available, it was essential to have an alternative route for communication, from managerial staff to all nurses and the last of the paramedical staff. Regular clear communication from the management proved critical for allowing essential patient care to continue
IT backup	This refers to having a backup for all essential information, having consistent parameter requirements across different vendor hardware and software, and having a plan for recovery and restoration of normal operations once the software is operational
Plans to support staff in a crisis and to maintain personal and organizational resilience	

## Conclusions

Hospitals must be prepared for cyberattacks just like any other emergency. The massive cyberattack had different impacts on various clinical hospital activities. The restoration of EMR and radiology archiving modules was found to be the most significant factor that allowed the return to normal hospital work. A selective approach to the decision-making process is needed to facilitate the provision of adequate patient care in different wards. Simulation of computer shutdown may assist in preparation for this kind of disaster and to be better prepared. It is recommended that healthcare providers at all levels have an available protocol for quick adaptation.

## Summary points

In this paper, we describe the difficulties and lessons that should be shared for awareness and learning following a ransomware attack on a medical center that resulted in the complete shutdown of all computer systems.

Our study describes the challenges following a cyberattack, the steps taken, and how they influenced hospital recovery.

Healthcare systems at all levels should be aware of this threat and implement protocols once this catastrophic event occurs. The restoration of EMR, laboratory systems, and imaging modules was found to be the most significant factor that allowed the return to normal clinical hospital work.

## Data Availability

The raw data supporting the conclusions of this article will be made available by the authors, without undue reservation.
